# Association between the dietary index for gut microbiota and cardiometabolic multimorbidity: systemic immune-inflammation index and systemic inflammatory response index

**DOI:** 10.3389/fnut.2025.1591799

**Published:** 2025-06-05

**Authors:** Sihan Hu, Jiayuan Song, Hui Jiang, Baojian Wei, Hui Wang

**Affiliations:** ^1^School of Public Health, Guangxi Medical University, Nanning, China; ^2^Changchun University of Chinese Medicine, Changchun, China; ^3^School of Nursing, Shandong First Medical University and Shandong Academy of Medical Sciences, Taian, China

**Keywords:** dietary index for gut microbiota, cardiometabolic multimorbidity, systemic immune-inflammation index, systemic inflammatory response index, NHANES

## Abstract

**Objective:**

This study targets to investigate the connection between the possibility of Cardiometabolic Multimorbidity (CMM) and the Dietary Index for Gut Microbiota (DI-GM), paying special attention to the mediating roles of systemic inflammatory markers, specifically the Systemic Immune-Inflammation Index (SII) and the Systemic Inflammatory Response Index (SIRI).

**Methods:**

A cross-sectional study was conducted involving 17,388 eligible participants from the National Health and Nutrition Examination Survey (NHANES) spanning from 2007 to 2020. Weighted logistic regression model was employed to assess the correlation between DI-GM, SII, SIRI, and CMM. Additionally, restricted cubic spline (RCS) regression model was utilized to investigate any potential nonlinear relationships between these variables and the risk of CMM. The degree to which SII and SIRI mediated the link between CMM and DI-GM was assessed using mediation analysis. Additionally, sensitivity and subgroup analyses were conducted to confirm the results.

**Results:**

A lower risk of CMM was markedly correlated with higher DI-GM scores (OR = 0.94, 95% CI: 0.91–0.98, *p* = 0.001). An elevated risk of CMM was markedly linked to higher levels of Ln-SII and Ln-SIRI (OR = 1.45, 95% CI: 1.28–1.65, *p* < 0.001; OR = 1.87, 95% CI: 1.69–2.07, *p* < 0.001). Higher education levels were associated with a stronger protective effect of DI-GM on CMM, according to subgroup analysis (*P* for interaction < 0.05). SII and SIRI, which accounted for 8.3 and 18.1% of the total effect, respectively, partially mediated the link between DI-GM and CMM (*p* < 0.001). Sensitivity analysis proved the stability of the findings.

**Conclusion:**

According to the study’s findings, DI-GM could mitigate the danger of CMM. Reduced systemic inflammation acted as a partial mediating factor in this connection. These findings highlight the mechanisms of gut microbiota to mitigate the danger of CMM from a nutritional perspective. This offers insightful information for clinical CMM therapy and prevention.

## 1 Introduction

Cardiometabolic multimorbidity (CMM) is defined by the concurrent presence of several cardiometabolic disorders (CMDs), such as hypertension, diabetes, coronary artery disease (CAD), and stroke (1). Globally, there has been a notable growth in the frequency of CMM. In the United States, for example, the prevalence of CMM climbed from 9.4% in 1999 to 14.4% in 2018 (2). Similarly, data from the China Health and Retirement Longitudinal Study (CHARLS) revealed that, among 7,909 participants who were free of CMM in 2011, 2,501 individuals had developed CMM by 2020 (3). The high prevalence of CMM not only poses serious health risks for patients but also markedly raises the risk of mortality from all causes (4). Specifically, individuals with CMM experience a reduction in life expectancy of more than 10 years by the age of 60 (5). Therefore, an in-depth investigation into the pathogenesis and intervention strategies of CMM is of great significance for improving patient outcomes and alleviating the public health burden.

Recently, the gut microbiota has emerged as a vital part of the human microbiome (6, 7). It plays crucial roles in maintaining intestinal barrier function, modulating the immune system, synthesizing vitamins and short-chain fatty acids and participating in metabolism as well as nutrient absorption (8).

Prior research has highlighted the link between gut microbiota and CMM. The gut microbiota composition varied significantly between diabetic and non-diabetic groups, with the non-diabetic group having more (SCFA), −generating bacteria (9). And the gut microbiota structure of CMM patients has undergone significant changes, which may manifest as alterations in microbial diversity and specific bacterial groups (10–12). Diet not only provides energy and nutrients but also profoundly affects the host’s metabolic health through altering the gut microbiota’s composition and function (10). Recently, Kase and his colleagues developed a Dietary Index for Gut Microbiota (DI-GM) to evaluate diet quality for a healthy gut microbiota (13). Compared to traditional dietary indices such as the Healthy Eating Index (HEI) and the Mediterranean Diet Score (MDS), the DI-GM focuses specifically on indicators such as gut microbiota diversity, SCFA levels, and the ratios of specific bacterial phyla. This targeted approach allows the DI-GM to more accurately reflect the impact of diet on gut microbiota regulation, demonstrating greater specificity for gut health (13). Nevertheless, investigation into the connection between DI-GM and CMM is scarce.

Inflammation is crucial in the development and progression of cardiovascular metabolic Syndrome. Novel inflammatory markers, such as systemic-inflammation index (SII) and systemic inflammatory response index (SIRI), have demonstrated predictive value across various chronic diseases (14, 15). In addition, chronic inflammation and gut microbiota seem to be in an interactive state, where inflammation can affect gut microbiota disorder and gut microbiota can regulate inflammation (16–18). They jointly participate in the pathogenesis of CMM. To sum up, we investigated the connection of CMM and DI-GM., utilizing NHANES data and probing into the possible mediating effects of the SII and SIRI.

## 2 Methods

### 2.1 The design for the study

**Figure 1:**
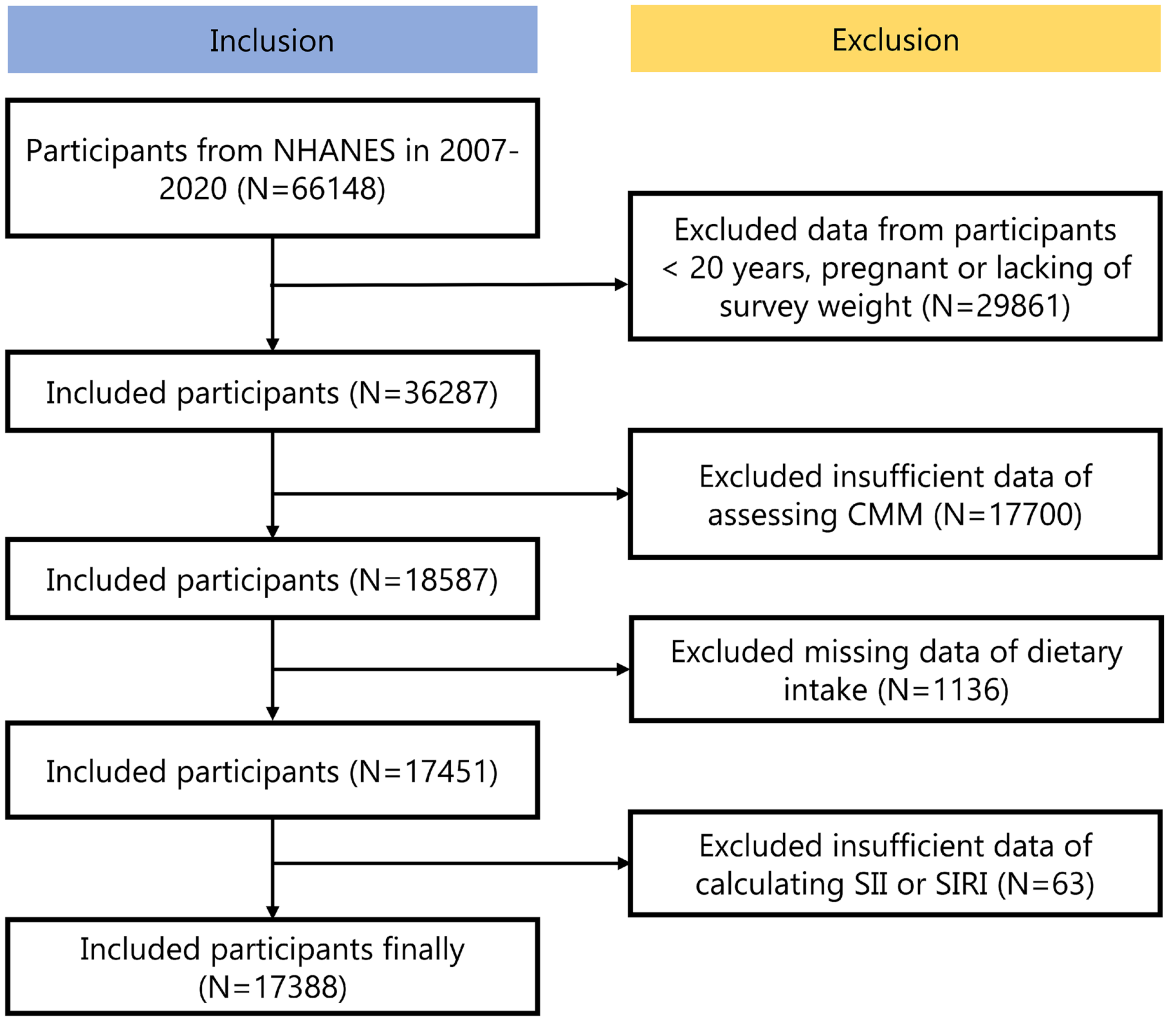
Participants’ screening process.

The NHANES is a national examination that collects comprehensive data on nutrition and health across the United States population (19). The dataset was publicly available and has been collected following ethical guidelines, including securing informed consent from all participants. The experimental designs and associated NHANES data were available on a publicly accessible platform: www.cdc.gov/nchs/nhanes/. Each methodological approach adhered strictly to the pertinent ethical standards and regulations. We analyzed the NHANES dataset spanning from 2007 to 2020, initially including 66,148 participants. Exclusions were made for 29,861 participants that were < 20 years, pregnant or lacking of survey weight, 17,700 participants with insufficient data of assessing CMM, 1136 participants with missing data of dietary intake and 63 participants with insufficient data of calculating SII or SIRI. Ultimately, the total was 17,388 participants (Figure 1).

### 2.2 Calculation of the dietary index for gut index microbiota

For the NHANES study, dietary information was collected using a 24-h recall approach. Each participant underwent two interviews conducted. The first interview took place in person, while the second one took place over the phone a few days later. Considering the greater accuracy of in-person data collection, we chose to use only the information gathered during the initial interview for our analysis (20).

Ten of the 14 different food or nutritional components that make up the DI-GM are considered beneficial to gut microbial multiplicity, whereas the remaining 4 are considered detrimental. Supplementary Table 1 provided a detailed list of meal kinds and computation techniques. The beneficial gut microbiota score (BGMS) and the unfavorable gut microbiota score (UGMS) are components of the overall DI-GM score, which runs from 0 to 13 (21). Four groups were also created from the overall scores: 0–3, 4, 5, and ≥ 6 points (22).

### 2.3 Definition of CMM

The CMDs encompassed hypertension, diabetes, CAD, and stroke (23). Diabetes was identified based on any of the following criteria: (1) a history of the condition, (2) current use of medications that lower blood glucose levels, or (3) a fasting plasma glucose level of at least 126 mg/dL or an HbA1c level of at least 6.5%. Hypertension was determined by: (1) a history of the condition, (2) the use of antihypertensive medications, or (3) a blood pressure reading of at least 140/90 mm Hg for systolic or diastolic pressure. Stroke and CAD were identified through self-reported clinical diagnoses. If a person had two or more CMDs, they were considered to have CMM.

### 2.4 Inflammatory index

Complete blood count on blood samples was detected in the mobile examination center (MEC). The following formulas were used to determine SII and SIRI values (24).

SII = (platelet count × neutrophil count)/lymphocyte count,

SIRI = (neutrophil count × monocyte count)/lymphocyte count.

### 2.5 Covariates

Covariates consisted of sociodemographic and life behavior variables (25). Please refer to Supplementary Table 2 for detailed classification for covariates.

### 2.6 Statistical analysis

We employed MEC examination weights to accurately reflect the U. S. population in this study (26). Weighted t-tests were used to evaluate continuous data, which were given as weighted mean ± standard deviation (SD). Weighted chi-square tests were implemented to assess categorical variables, which were represented as number and weighted proportions. Missing covariates were processed by “MICE” package using multiple imputation (27). We Ln-transformed SII and SIRI to resolve their skewness (28).

Using weighted logistic regression models, we first evaluated the link between DI-GM, SII, and SIRI and the probability of CMM, calculating odds ratios (OR) with 95% confidence intervals (CI). These variables were analyzed both continuously and categorically across three models: Model 1 was left uncorrected, Model 2 corrected for demographic characteristics, while Model 3 took into account all covariates (29). To explore potential non-linear relationships between DI-GM, SII, SIRI, and CMM risk, we constructed restricted cubic spline (RCS) regression models, optimizing the number of knots based on the minimum Akaike information criterion (AIC) (30). Subgroup analyses followed to evaluate interaction effects between DI-GM and covariates on CMM risk, using likelihood ratio tests. Finally, mediation analysis was applied to evaluate whether the inflammation index played a mediating function. Mediation analysis tests the total effect (TE), direct effect (DE), and indirect effect (IE) between variables. We established 1,000 times repeated sampling to improve the accuracy of the results (31). Moreover, we carried out a sensitivity analysis and exclude participants with extreme energy intake and verify the results’ resilience (32). R software (version 4.4.1) was used for all analyses. The criterion for statistical significance was set at *P* less than 0.05.

## 3 Results

### 3.1 Study objects characteristics

Our study encompassed a total of 17,388 study objects, comprising 8,662 males (49.8%) and 8,726 females (50.2%). They were categorized into two groups: the non-CMM group, which consisted of 12,640 individuals, and the CMM group, with 4,748 individuals. Table 1 presented the characteristics of the participants. Across all variables examined, statistically significant differences were noted between the non-CMM and CMM groups (All *p* < 0.05).

**Table 1:** Characteristics of participants grouped by CMM in NHANES 2007–2020.

Variables	Total	Non-CMM group	CMM group	*P*
	17,388	12,640	4,748	
Age, n (%)				< 0.001
20–39	4,827 (31.2)	4,642 (38.2)	185 (4.8)	
40–59	5,905 (38.1)	4,555 (39.2)	1,350 (33.9)	
≥ 60	6,656 (30.8)	3,443 (22.6)	3,213 (61.3)	
Gender, n (%)				< 0.001
Male	8,662 (49.8)	6,174 (49)	2,488 (52.7)	
Female	8,726 (50.2)	6,466 (51)	2,260 (47.3)	
Race, n (%)				< 0.001
Mexican American	2,652 (8.5)	1990 (8.9)	662 (7.1)	
Other Hispanic	1891 (5.9)	1,379 (6)	512 (5.5)	
Non-Hispanic White	7,091 (67.1)	5,290 (67.7)	1801 (64.7)	
Non-Hispanic Black	3,790 (10.7)	2,421 (9.6)	1,369 (15.2)	
Other Race—Including Multi-Racial	1964 (7.7)	1,560 (7.7)	404 (7.6)	
Education, n (%)				< 0.001
Less than high school	4,347 (16)	2,802 (14.3)	1,545 (22.4)	
High school grad/GED or equivalent	3,962 (23.5)	2,803 (22.4)	1,159 (27.3)	
Higher than high school	9,079 (60.6)	7,035 (63.3)	2044 (50.3)	
PIR, n (%)				< 0.001
≤1.3	5,694 (22.4)	3,994 (21.5)	1700 (26)	
1.3–3.5	6,610 (36)	4,688 (34.9)	1922 (40)	
> 3.5	5,084 (41.6)	3,958 (43.6)	1,126 (34)	
Marital status, n (%)				< 0.001
Married/living with partner	10,360 (63.9)	7,604 (64)	2,756 (63.3)	
Widowed/divorced/separated	4,106 (19.4)	2,543 (17.1)	1,563 (28.3)	
Never married	2,922 (16.7)	2,493 (18.9)	429 (8.3)	
BMI, n (%)				< 0.001
< 5 kg/m^2^	4,466 (27)	3,856 (31.1)	610 (11.5)	
25–30 kg/m^2^	5,573 (32.1)	4,252 (33.9)	1,321 (25.5)	
≥ 30 kg/m^2^	7,349 (40.9)	4,532 (35)	2,817 (63)	
Smoking status, n (%)				< 0.001
Non smokers	9,466 (54.2)	7,178 (56.2)	2,288 (46.6)	
Former smokers	4,529 (27.2)	2,847 (24.4)	1,682 (37.7)	
Current smokers	3,393 (18.6)	2,615 (19.3)	778 (15.7)	
Drinking status, n (%)				< 0.001
Non drinkers	6,349 (29.5)	3,989 (25.7)	2,360 (43.6)	
Moderate drinkers	9,977 (63.3)	7,775 (66.2)	2,202 (52.2)	
Heavy drinkers	1,062 (7.3)	876 (8.1)	186 (4.2)	
Physical activity, n (%)				< 0.001
Yes	10,119 (63.6)	8,040 (67.9)	2079 (47.5)	
No	7,269 (36.4)	4,600 (32.1)	2,669 (52.5)	
Daily energy intake, mean (SD)	2146.58 (967.23)	2199.50 (975.52)	1949.28 (909.02)	< 0.001
DI-GM, mean (SD)	4.59 (1.63)	4.63 (1.63)	4.45 (1.65)	< 0.001
DI-GM group, n (%)				< 0.001
0–3	4,490 (24.5)	3,157 (23.7)	1,333 (27.7)	
4	4,578 (24.6)	3,307 (24.4)	1,271 (25.2)	
5	3,931 (23.1)	2,891 (23.3)	1,040 (22.4)	
*≥* 6	4,389 (27.8)	3,285 (28.6)	1,104 (24.7)	
SII, mean (SD)	526.17 (339.75)	507.15 (312.93)	597.10 (417.55)	< 0.001
SIRI, mean (SD)	1.23 (0.89)	1.14 (0.77)	1.57 (1.16)	< 0.001

### 3.2 Associations between DI-GM and CMM

The findings of the weighted logistic regression analysis examining the connection between DI-GM scores and CMM were presented in Table 2. A 7% decrease in CMM risk was associated with a 1-point increase in DI-GM in the original model (Model 1) (OR: 0.93, 95% CI: 0.91, 0.96, *p* < 0.001). Further adjustment for all factors in Model 3 maintained a significant association, with a 6% reduction in CMM risk with per 1-point increase in DI-GM (OR: 0.94, 95% CI: 0.91, 0.98, *p* = 0.001). After controlling for all factors, in categorical analysis the likelihood of CMM was 24% lower for individuals that had a DI-GM score greater than 6 than for those in the lowest DI-GM score group (OR: 0.76, 95% CI: 0.64, 0.89, *p* < 0.001). Trend analysis showed a strong inverse correlation (*P* for trend < 0.001) between CMM likelihood and DI-GM scores.

**Table 2:** The associations between DI-GM and CMM in weighted logistic regression models.

	Model 1		Model 2		Model 3	
	OR (95% CI)	*P*	OR (95% CI)	*P*	OR (95% CI)	*P*
DI-GM						
Per 1-point increment	0.93 (0.91, 0.96)	< 0.001	0.91 (0.88, 0.94)	< 0.001	0.94 (0.91, 0.98)	0.001
DI-GM group						
0–3	Reference		Reference		Reference	
4	0.88 (0.78, 1.00)	0.056	0.87 (0.75, 1.02)	0.079	0.92 (0.78, 1.08)	0.279
5	0.82 (0.71, 0.96)	0.012	0.82 (0.69, 0.98)	0.029	0.88 (0.73, 1.06)	0.175
*≥* 6	0.74 (0.65, 0.83)	< 0.001	0.66 (0.57, 0.77)	< 0.001	0.76 (0.64, 0.89)	< 0.001
*P* for trend	< 0.001		< 0.001		< 0.001	
BGMS	0.91 (0.88, 0.94)	< 0.001	0.92 (0.89, 0.96)	< 0.001	0.96 (0.92, 1.01)	0.085
UGMS	0.99 (0.95, 1.03)	0.569	0.91 (0.87, 0.96)	< 0.001	0.90 (0.84, 0.96)	< 0.001

Model 1 was not adjusted for any covariate. Model 2 was adjusted for age, gender, race, education, PIR, marital status. Model 3 was adjusted for all covariates.

Interestingly, we found that BGMS was marginal significantly with a lower CMM hazard (OR: 0.96, 95% CI: 0.92, 1.01, *p* = 0.085), whereas UGMS was obviously with a lower CMM hazard (OR: 0.90, 95% CI: 0.84, 0.96, *p* < 0.001) in Model 3.

**Figure 2:**
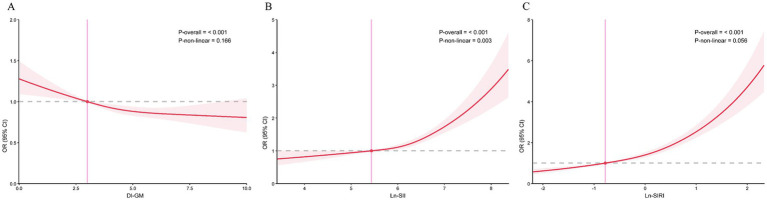
The correlations of DI-GM, SII, and SIRI with CMM in RCS regression models. **(A)** DI-GM, **(B)** SII, **(C)** SIRI. Models were adjusted for all covariates.

Following this, we constructed RCS model with 3 knots to investigate the potential non-linear correlation between CMM and DI-GM (Figure 2A). The model indicated that, after accounting for all covariates, there was a linear negative correlation of DI-GM with the risk of CMM, with no significant non-linear effect observed (*P* for non-linear > 0.05).

### 3.3 Associations between two inflammatory index and CMM

Table 3 highlighted that Ln-SIRI and Ln-SII were significantly connected with the probability of CMM after controlling for all variables. A 45% increased risk of CMM was linked to every unit increase in Ln-SII (OR: 1.45, 95% CI: 1.28, 1.65, *p* < 0.001), and an 87% increased risk of CMM was linked to each unit increase in Ln-SIRI (OR: 1.87, 95% CI: 1.69, 2.07, *p* < 0.001). In categorical analyses, compared to Q1 of Ln-SII, the objects with Q3 and Q4 of Ln-SII had a greater danger of CMM, and the objects with Q2, Q3 and Q4 of ln-SIRI had a higher risk of CMM (All ORs > 1, *p* < 0.05). In addition, the trend test identified a strong positive correlation between the probability of CMM and two inflammatory markers (Both *P* for trend < 0.001).

**Table 3:** The associations of SII, SIRI, and CMM in weighted logistic regression models.

	Model 1		Model 2		Model 3	
	OR (95% CI)	*P*	OR (95% CI)	*P*	OR (95% CI)	*P*
Ln-SII						
Per 1-unit increment	1.61 (1.44, 1.81)	< 0.001	1.56 (1.38, 1.75)	< 0.001	1.45 (1.28, 1.65)	< 0.001
Q1	Reference		Reference		Reference	
Q2	1.03 (0.90, 1.18)	0.695	1.12 (0.96, 1.31)	0.145	1.06 (0.90, 1.25)	0.485
Q3	1.32 (1.12, 1.55)	0.001	1.39 (1.17, 1.66)	< 0.001	1.24 (1.04, 1.49)	0.019
Q4	1.79 (1.54, 2.07)	< 0.001	1.76 (1.48, 2.09)	< 0.001	1.56 (1.30, 1.88)	< 0.001
*P* for trend	< 0.001		< 0.001		< 0.001	
Ln-SIRI						
Per 1-unit increment	2.35 (2.16, 2.55)	< 0.001	2.03 (1.85, 2.23)	< 0.001	1.87 (1.69, 2.07)	< 0.001
Q1	Reference		Reference		Reference	
Q2	1.28 (1.10, 1.48)	0.002	1.37 (1.16, 1.62)	< 0.001	1.27 (1.07, 1.52)	0.008
Q3	1.75 (1.54, 1.98)	< 0.001	1.73 (1.50, 1.98)	< 0.001	1.48 (1.29, 1.71)	< 0.001
Q4	3.58 (3.17, 4.05)	< 0.001	3.05 (2.66, 3.51)	< 0.001	2.58 (2.23, 3.00)	< 0.001
*P* for trend	< 0.001		< 0.001		< 0.001	

Model 1 was not adjusted for any covariate. Model 2 was adjusted for age, gender, race, education, PIR, marital status. Model 3 was adjusted for all covariates.

As illustrated by Figures 2B,C, RCS regression models exhibited a linear positive correlation between Ln-SIRI and CMM (*P* for non-linear > 0.05) and a nonlinear positive correlation of Ln-SII and CMM (*P* for non-linear < 0.05).

### 3.4 Subgroup analyses

**Figure 3:**
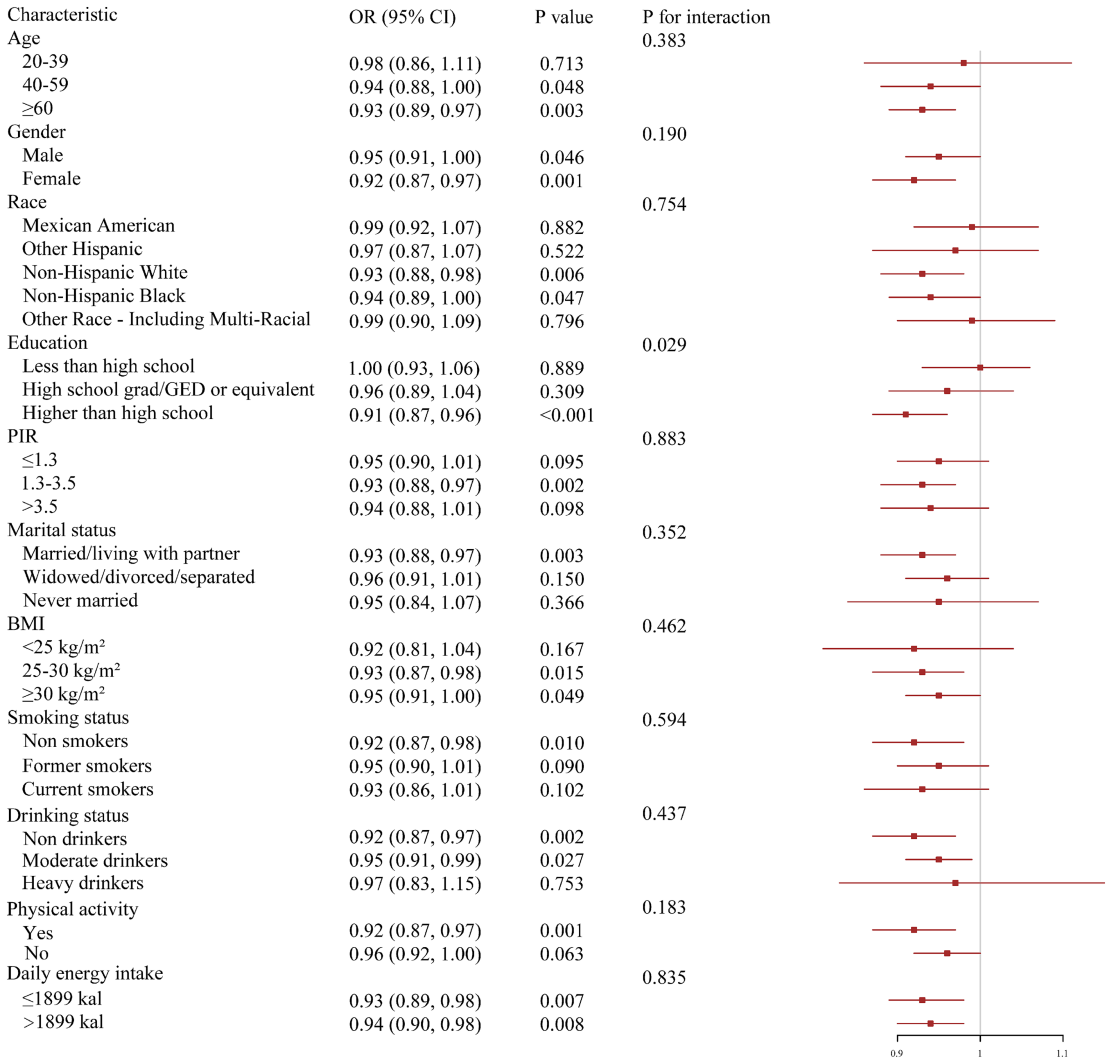
Subgroup analysis of associations of DI-GM and CMM. Models were adjusted for all covariates.

We explored the correlations of DI-GM with CMM according to different groups of covariates (Figure 3). In the vast majority of groups, DI-GM still demonstrated its beneficial effects, consistent with the analysis results above. We found that DI-GM and education level had an obvious interaction effect on CMM (*P* for interaction < 0.05). The impact of DI-GM on CMM is obvious in participants with education level higher than high school, while no significant effect of DI-GM was found in the other two subcategories.

### 3.5 The mediating function of inflammation index

**Figure 4:**
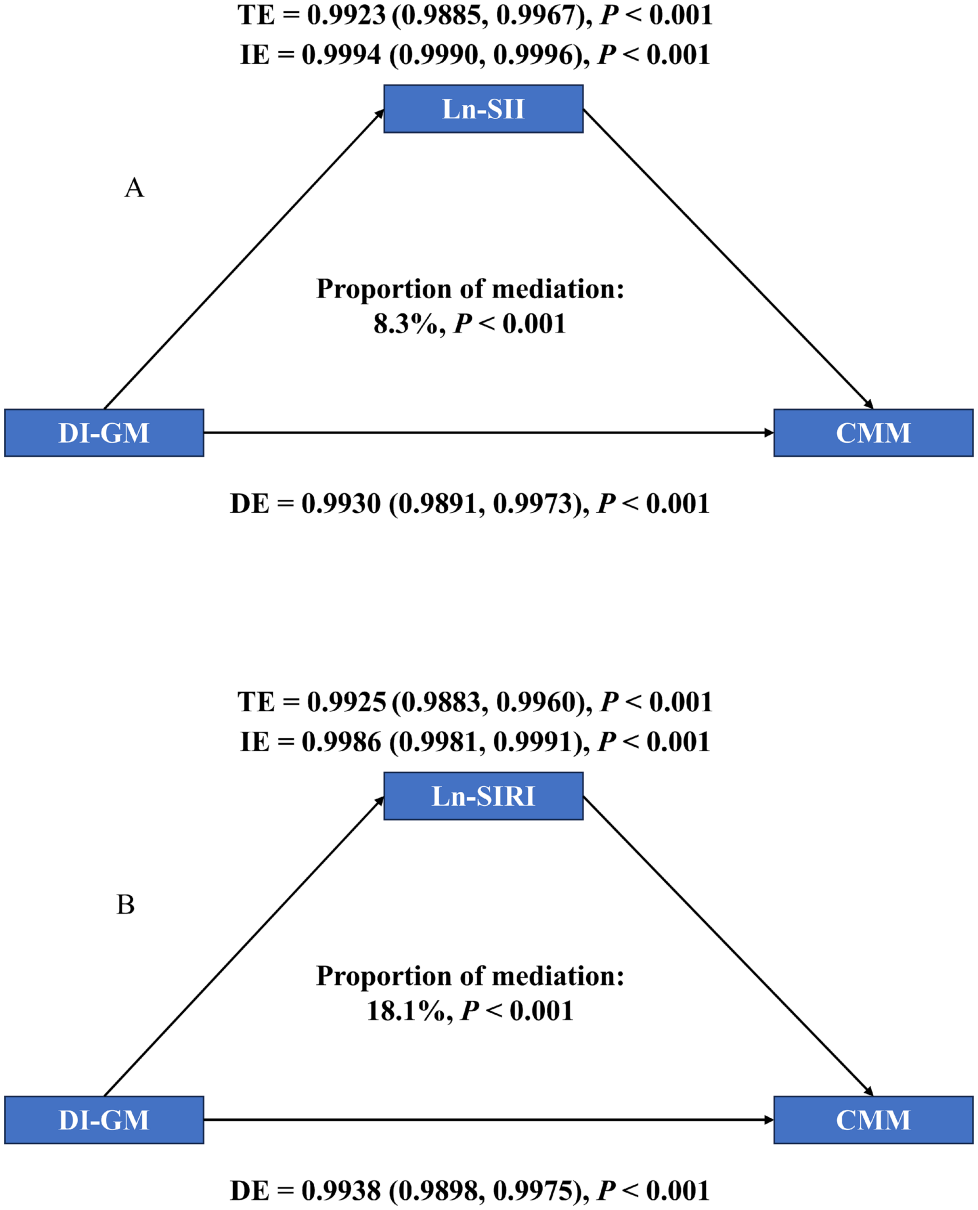
Mediation analysis of exploring the mediating effect of SII and SIRI. **(A)** SII, **(B)** SIRI. Models were adjusted for all covariates.

The mediating influence of SII and SIRI was investigated using mediation analyses (Figure 4). TE, IE, and DE were all obvious in two mediation models (*p* < 0.001). The connections between DI-GM and CMM were specifically mediated by Ln-SII and Ln-SIRI, which accounted for 8.3 and 18.1% of the corresponding association, respectively (*p* < 0.001). According to these results, DI-GM decreased the risk of CMM by reducing inflammation.

### 3.6 Sensitivity analysis

We excluded participants with extreme energy intake and further assessed the association between the DI-GM and CMM (Supplementary Table 3). The significant correlation between higher DI-GM and lower CMM risk did not change.

## 4 Discussion

In this study, we investigated the link of DI-GM with CMM, and the mediating functions of SII and SIRI. Weighted logistic regression models and RCS analysis consistently indicated that CMM risk may be decreased by DI-GM, but it may be increased by SII and SIRI. Mediating analysis confirmed that the reduction of inflammation is a mechanism by which DIGM reduces the risk of CMM. Sensitivity analysis supported the robustness of our research results. Our results emphasized the latent capacity of DI-GM to lower the risk of CMM and the underlying mechanisms of inflammation, offering fresh approaches to clinical prevention and care of CMM and having important public health implications.

Our findings demonstrated alignment with prior investigations examining the associations of gut microbiota with individual CMD risks, while extending evidence to the pathophysiological spectrum of multimorbidity clustering. A negative connection between DI-GM and diabetes risk was found by Wu et al., indicating that maintaining DI-GM lowers the chance of developing diabetes (10). Separately, Liu et al. illustrated that elevated DI-GM scores and indices favorable to Gut microbiota has been connected with a decreased likelihood of stroke, especially among individuals aged 30 and older (21). Moreover, another study indicated that there is an adverse relationship between DI-GM and the prevalence of metabolic dysfunction-associated fatty liver disease (MAFLD) in the United States (33).

The gut microbiota and health are closely related to cardiovascular and metabolic health. The gut microbiota exhibits excellent anti-inflammatory and antioxidant effects, and inflammation and oxidative stress are important factors affecting cardiac metabolism (34–36). In addition, metabolites were produced by the intestinal microbiota, including trimethylamine N-oxide (TMAO), SHFA, and phenylacetylglutamine, might either enhance or suppress the development of cardiovascular disease (37, 38). In addition, the metabolites of probiotics exhibited angiotensin-converting enzyme (ACE) inhibitory properties, leading to their anti-hypertensive effects (39). The evaluation of DI-GM includes 14 foods or nutrients that are closely related to gut health and cardiovascular metabolic health. A clinical trial showed that the avocado supplementation group showed a significant increase in *Faecalibacterium prausnitzii* and *AF16_15* bacteria (40). *Faecalibacterium prausnitzii* alleviates inflammation and strengthens intestinal machinery and mucosal barrier, leading to lower plasma lipopolysaccharide level and anti-atherosclerosis (41). In addition, avocado is rich in unsaturated fatty acids (UFA), which help promote blood lipid health and reduce the risk of metabolic diseases (42, 43). One cruciferous vegetable that is high in isothiocyanates is broccoli. These substances increase myrosinase activity in the colon and cecum, and they lower the risk of cardiovascular disease (CVD) by acting as antioxidants and anti-inflammatory agents (44–46). The annual consumption of chickpeas in the United States is increasing year by year. A meta-analysis showed that because of their high fiber and protein content, low starch digestion, and hormonal impacts, chickpeas may help regulate blood sugar levels (47). Coffee and tea are rich in caffeine, which may reduce the incidence of CMD by affecting gut microbiota, increasing energy expenditure, and improving lipid metabolism (48–50). In addition, cranberries are rich in unique phytochemicals, including anthocyanins, flavonoids, and phenolic acids (51). The metabolites of anthocyanins have shown good effects in improving gut microbiota composition, anti-inflammatory and antioxidant properties (52). Flavonoids are present in a large number of plant-based foods. It can promote the production of beneficial gut microbiota, inhibit pathogen growth and endotoxin production to maintain intestinal immune homeostasis. Fermented dairy products contain probiotics, including *Streptococcus thermophilus* and *Lactobacillus delbrueckii*, which can lower the risk of diabetes and CVD by enhancing the digestive tract’s internal and exterior health (53, 54). In addition, the favorable impacts of dietary fiber, soybeans, and whole grains on cardiac metabolism can also be determined (55–57). Dietary fiber also can actively regulate the gut microbiota (58). Soybeans are rich in soybean isoflavones (Sis). SIs can enhance intestinal secretion capacity, regulate inflammatory signaling pathways, affect intestinal barrier function and regulates glucose homeostasis and lipid metabolism (59, 60).

On the contrary, the remaining four components seem to be detrimental to CMM. Red meat contains N-nitroso compounds, heterocyclic amines, and heme, which can cause imbalance dysbiosis of the gut microbiota (61). More importantly, meta-analysis on red meat reveals adverse effects on CVD regardless of whether red meat is processed or not (62, 63). Compared to whole grains, refined grains are obtained after grain processing, resulting in significant loss of nutrients such as dietary fiber, B vitamins, magnesium, iron, etc. Therefore, high intake of refined grains may lead to lower intake of these nutrients, which is detrimental to metabolic diseases (64, 65). Finally, the adverse effects of high-fat intake on cardiac metabolism have been widely recognized (66, 67). In summary, these 14 foods or nutrients are interconnected with intestinal health and lower likelihood of CMDs through anti-inflammatory, antioxidant, and lipid-lowering pathways. For the first time, we have discovered that DI-GM decreases the risk of inflammation and further CMM in the populace at large, which confirms the conclusions of the above literature.

Subgroup analysis results showed that among individuals with education levels higher than high school, DI-GM had a greater reducing effect on CMM. This may be related to people with more education are more concerned about eating healthily with a high content of dietary fiber, unsaturated fatty acids as well as antioxidants, and significantly decrease inflammation and cardiovascular disease risk (68, 69).

Our research is distinguished by several key strengths. Firstly, we have conducted the pioneering investigation into the relationship between the DI-GM and CMM, thereby providing innovative perspectives on the nutritional approaches to preventing CMM. Secondly, the robustness of our findings is bolstered by the application of a variety of statistical techniques. Thirdly, we have elucidated the mediation effects, shedding light on the possible underlying mechanisms of this relationship.

Notwithstanding these strengths, the study is subject to constraints. Foremost, the cross-sectional study means that data were collected at a specific moment in time, which precludes the causality between exposure and outcomes (70). Secondly, the research population is the American population, so it is necessary to be cautious when extrapolating the research conclusions to other populations. Thirdly, our analysis focused solely on the effects of SII and SIRI in the DI-GM-CMM relationship, potentially overlooking the role of other inflammatory biomarkers. Finally, the DI-GM has its own limitations. There is a lack of clear definition for beneficial/harmful gut microbiota, and the indicators used in different studies are also inconsistent.

## 5 Conclusion

DI-GM that reflects gut microbiota could significantly reduce the occurrence of CMM. Inflammation may be one of the factors that underlie this advantageous effect. Our study elucidates the underlying mechanisms through which the gut microbiota modulates cardiac metabolism, offering novel insights for the prevention and management of CMM.

## Data Availability

The original contributions presented in the study are included in the article/Supplementary material, further inquiries can be directed to the corresponding authors.

## Glossary

AIC: Akaike information criterion
BMI: body mass index
CAD: coronary artery disease
CHARLS: China Health and Retirement Longitudinal Study
CMD: cardiometabolic diseases
CMM: Cardiometabolic Multimorbidity
CVD: Cardiovascular disease
DE: direct effect
DI-GM: Dietary Index for Gut Microbiota
HEI: Healthy Eating Index
IE: indirect effect
MAFLD: metabolic- dysfunction related fatty liver disease
MEC: mobile examination center
NHANES: National Health and Nutrition Examination Survey
OR: Odds ratios
CI: confidence intervals
PIR: poverty-to-income ratio
RCS: restricted cubic spline
SII: systemic immune inflammation index
SIRI: systemic inflammatory response index
TE: total effect
TMAO: trimethylamine N-oxide
SCFA: short-chain fatty acid
