# Supplementary material for: Association between the dietary index for gut microbiota and cardiometabolic multimorbidity: systemic immune-inflammation index and systemic inflammatory response index
Source: Front Nutr. 2025 Jun 5;12:1591799. doi: 10.3389/fnut.2025.1591799 (PMC12176575; doi:10.3389/fnut.2025.1591799)
Supplement: Supplementary file 1 [file Supplementary_file_1.docx]

Supplementary Table 1. Components and scoring criteria of DI-GM in NHANES.

| Components of  DI-GM | Food items included in NHANES | Scoring criteria |
| --- | --- | --- |
| Beneficial to gut microbiota | Avocados, Broccoli, Chickpeas, Coffee, Cranberries, Fermented dairy (including yogurt, cheese, kefir, sour cream, buttermilk), Fiber, Soybean (including Soy milk, Tofu), Whole grains | Score 1 - Consumption ≥ sex-specific median  Score 0 - Otherwise |
| Unfavorable to gut microbiota | Refined grains, Processed meat, Red meat | Score 0 - Consumption ≥ sex-specific median  Score 1 - Otherwise |
|  | High-fat diet (% energy) | Score 0 - Consumption ≥ 40%  Score 1 - Otherwise |

Supplementary Table 2. Classification and definition of covariates.

| Covariate Category | Covariate Name | Categories |
| --- | --- | --- |
| Sociodemographic Variables | Gender | Male, Female |
|  | Age | 20–39 years, 40–59 years, ≥60 years |
|  | Race | Mexican American, Other Hispanic, Non-Hispanic White, Non-Hispanic Black, Other Race (including multi-racial) |
|  | Educational Level | Less than high school, High school graduate/GED or equivalent, Higher than high school |
|  | Household Poverty-to-Income Ratio (PIR) | ≤1.3, 1.3–3.5, >3.5 |
|  | Marital Status | Married/Living with a partner, Widowed/Divorced/Separated, Never married |
| Life Behavior Variables | Body Mass Index (BMI) | <25 kg/m², 25-30 kg/m², ≥30 kg/m² |
|  | Smoking Status | Nonsmokers, Former smokers, Current smokers |
|  | Drinking Status | Nondrinkers (no alcohol), Moderate drinkers (≤2 drinks/day for male, ≤1 drink/day for female), Heavy drinkers (>2 drinks/day for male, >1 drink/day for female) |
|  | Physical Activity | Achieved 600 metabolic equivalent (MET) minutes per week |
|  | Daily Energy Intake | From the dietary survey on the first day |

Supplementary Table 3. The associations DI-GM and CMM in weighted logistic regression models（excluded daily energy intake < 500 or > 5000 kcal/day for females, and < 500 or > 8000 kcal/day for males）.

|  | Model 1 |  |  | Model 2 |  |  | Model 3 |  |
| --- | --- | --- | --- | --- | --- | --- | --- | --- |
|  | OR (95% CI) | *P* |  | OR (95% CI) | *P* |  | OR (95% CI) | *P* |
| DI-GM |  |  |  |  |  |  |  |  |
| Per 1 point increment | 0.93 (0.91, 0.96) | < 0.001 |  | 0.92 (0.89, 0.95) | < 0.001 |  | 0.95 (0.92, 0.99) | 0.008 |
| DI-GM group |  |  |  |  |  |  |  |  |
| 0-3 | Reference |  |  | Reference |  |  | Reference |  |
| 4 | 0.88 (0.76, 1.01) | 0.059 |  | 0.88 (0.74, 1.04) | 0.122 |  | 0.93 (0.78, 1.10) | 0.389 |
| 5 | 0.81 (0.70, 0.95) | 0.008 |  | 0.83 (0.69, 1.00) | 0.054 |  | 0.90 (0.74, 1.09) | 0.281 |
| *≥* 6 | 0.74 (0.66, 0.83) | < 0.001 |  | 0.68 (0.58, 0.80) | < 0.001 |  | 0.79 (0.67, 0.94) | 0.007 |
| *P* for trend | < 0.001 |  |  | < 0.001 |  |  | 0.005 |  |
| BGMS | 0.91 (0.88, 0.94) | < 0.001 |  | 0.93 (0.89, 0.97) | 0.001 |  | 0.97 (0.92, 1.01) | 0.156 |
| UGMS | 0.99 (0.95, 1.03) | 0.528 |  | 0.92 (0.88, 0.96) | < 0.001 |  | 0.91 (0.86, 0.98) | 0.008 |

Model 1 was not adjusted for any covariate.

Model 2 was adjusted for age, gender, race, education, PIR, marital status.

Model 3 was adjusted for age, gender, race, education, PIR, marital status, BMI, smoking status, drinking status, physical activity and daily energy intake.
